# Cardiovascular protection of YiyiFuzi powder and the potential mechanisms through modulating mitochondria-endoplasmic reticulum interactions

**DOI:** 10.3389/fphar.2024.1405545

**Published:** 2024-06-24

**Authors:** Jingyi Ding, Ran Ji, Ziyi Wang, Yuzhi Jia, Tiantian Meng, Xinbin Song, Jing Gao, Qingyong He

**Affiliations:** ^1^ Department of Cardiology, Guang’anmen Hospital, China Academy of Chinese Medical Sciences, Beijing, China; ^2^ Department of Intensive Care Unit, Guang’anmen Hospital, China Academy of Chinese Medical Sciences, Beijing, China; ^3^ Department of Rehabilitation, Dongfang Hospital, Beijing University of Chinese Medicine, Beijing, China; ^4^ Graduate School, Henan University of Chinese Medicine, Zhengzhou, China

**Keywords:** cardiovascular disease, mitochondria, endoplasmic reticulum, Chinese herbal medicine, YiYiFuZi powder

## Abstract

Cardiovascular diseases (CVD) remain the leading cause of death worldwide and represent a major public health challenge. YiyiFuzi Powder (YYFZ), composed of Coicis semen and Fuzi, is a classical traditional Chinese medicine prescription from the *Synopsis of Golden Chamber* dating back to the Han Dynasty. Historically, YYFZ has been used to treat various CVD, rooted in Chinese therapeutic principles. Network pharmacology analysis indicated that YYFZ may exhibit direct or indirect effects on mitochondria-endoplasmic reticulum (ER) interactions. This review, focusing on the cardiovascular protective effects of Coicis semen and Fuzi, summarizes the potential mechanisms by which YYFZ acts on mitochondria and the ER. The underlying mechanisms are associated with regulating cardiovascular risk factors (such as blood lipids and glucose), impacting mitochondrial structure and function, modulating ER stress, inhibiting oxidative stress, suppressing inflammatory responses, regulating cellular apoptosis, and maintaining calcium ion balance. The involved pathways include, but were not limited to, upregulating the IGF-1/PI3K/AKT, cAMP/PKA, eNOS/NO/cGMP/SIRT1, SIRT1/PGC-1α, Klotho/SIRT1, OXPHOS/ATP, PPARα/PGC-1α/SIRT3, AMPK/JNK, PTEN/PI3K/AKT, β2-AR/PI3K/AKT, and modified Q cycle signaling pathways. Meanwhile, the MCU, NF-κB, and JAK/STAT signaling pathways were downregulated. The PERK/eIF2α/ATF4/CHOP, PERK/SREBP-1c/FAS, IRE1, PINK1-dependent mitophagy, and AMPK/mTOR signaling pathways were bidirectionally regulated. High-quality experimental studies are needed to further elucidate the underlying mechanisms of YYFZ in CVD treatment.

## 1 Introduction

Cardiovascular diseases (CVD) remain the leading global cause of mortality, presenting a major public health challenge (Perk, 2017; Luo F. et al., 2018). In 2022, approximately 775 million individuals worldwide were affected by CVD, contributing to over 19.8 million deaths (Mensah et al., 2023). Currently, there is compelling evidence indicating that the occurrence and progression of CVD are frequently associated with aberrant interactions between mitochondria and endoplasmic reticulum (ER) (Gao et al., 2020; Wang et al., 2021e).

Mitochondria serve as the cellular powerhouses, generating energy in the form of adenosine triphosphate (ATP) and playing crucial roles in many cellular processes. Meanwhile, the ER is vital for protein synthesis, folding, translocation, and maintaining calcium (Ca^2+^) homeostasis (Zhou et al., 2021). In recent years, the focus on regulating mitochondrial quality control (MQC) has opened new avenues for treating CVD (Chang et al., 2022; Chang et al., 2023a; Chang et al., 2023b; Chang et al., 2023c). This autonomic system safeguards cardiomyocytes during stress responses by inhibiting mitochondrial apoptosis, mitochondrial-related ER stress, and the mitochondrial-related unfolded protein response (UPR). As a result, it mitigates ischemia and hypoxia injuries in sinoatrial node cells (Chang et al., 2023a; Chang et al., 2024b) and normal cardiomyocytes (Chang et al., 2021; Chang et al., 2022; Chang et al., 2023b; Chang et al., 2024a), ultimately protecting cardiovascular health. It is evident that the roles of mitochondria and ER in cardiovascular health are intertwined, indicating an interconnected relationship rather than independent functions. These two organelles are intricately connected both structurally and functionally, forming specialized domains known as mitochondria-associated endoplasmic reticulum membranes (MAM) (Marchi et al., 2014; Szymanski et al., 2017; Gao et al., 2020), which are integral to fundamental biological processes. Abnormal MAM structure and mitochondria-ER interactions can lead to disruptions in mitochondrial structure, function, DNA, and biosynthesis (Zhu L. et al., 2020; Glanz et al., 2020) as well as altered lipid metabolism, impaired Ca^2+^ handling, ER stress, oxidative stress, inflammation, and apoptosis (Gao et al., 2020; Wang et al., 2021e; Luan et al., 2021). Dysfunction in both mitochondria and ER can contribute to the development and progression of various CVD, including heart failure (HF) (Liu et al., 1992; Lu et al., 2017), cardiac hypertrophy (Dromparis et al., 2013; Koyama et al., 2014; Wu Y. et al., 2016), atherosclerosis (Glanz et al., 2020), coronary heart disease (CHD) (Liu and Wu, 2022), and myocardial infarction (MI) (Liu et al., 1992; Wang N. N. et al., 2021).

**FIGURE 1:**
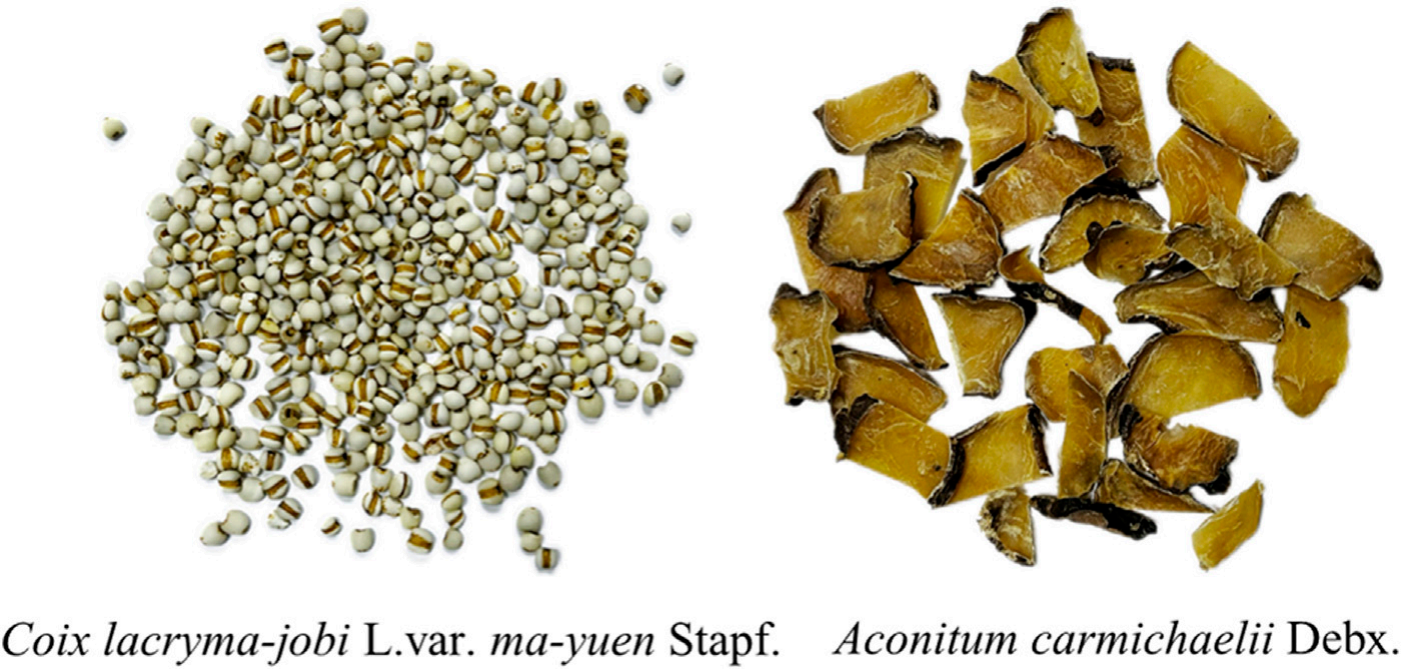
The processed raw materials of YiyiFuzi Powder.

Traditional Chinese medicine (TCM), an essential complementary and alternative medicine, has been used for thousands of years to treat various diseases with multitarget effects and reported clinical benefits (Lu et al., 2004; Meng et al., 2021; Lv S. et al., 2022; Liu Y. J. et al., 2022). YiyiFuzi Powder (YYFZ), composed of Coicis semen and Fuzi (Figure 1), is a classical prescription from the *Synopsis of Golden Chamber* by Zhang Zhongjing in the Han Dynasty, which historically relieved cardiothoracic pain. In TCM theory, Coicis semen removes dampness and eliminates blockages (Han et al., 2017; Luo Y. Y. et al., 2018), while Fuzi warms and strengthens Yang, relieves pain, and disperses cold (Commission, 2020). Combining Coicis semen and Fuzi can remove the paralysis of cold and dampness contributing to cardiothoracic pain (Yang et al., 2016). Additional benefits have been noted in unstable angina patients, including improvements in their clinical symptoms, signs, and electrocardiogram ischemia, as well as reductions in homocysteine levels (Wang, 2018). The formula also treats other systemic conditions such as rheumatoid arthritis (Zhang F. F. et al., 2023; Wang Y. Y. et al., 2023) and bronchial asthma (Yu Z. X. et al., 2011; Yu et al., 2016).

Network pharmacology analysis suggests that YYFZ may impact mitochondria-ER interactions (Wang, 2019; Wang Y. et al., 2023). Our study reviews the cardiovascular protective effects of YYFZ by directly regulating mitochondrial and ER function and indirectly influencing cardiovascular risk factors (such as lipid and glucose metabolism), oxidative stress, inflammatory responses, cellular apoptosis, and Ca^2+^ balance to impact the mitochondria-ER interactions. These findings provide a theoretical basis for the rational clinical application of the TCM formula YYFZ.

## 2 The cardiovascular protective activity of YYFZ

Modern studies have demonstrated the effectiveness of YYFZ in therapeutic applications for CVD, including chronic heart failure (CHF) (Wang Y. Y. et al., 2023), CHD (Wang, 2018), myocardial ischemia (Li et al., 2023), and myocarditis (Liu, 1991), with reported analgesic, anti-anginal, and cardiotonic effects (Wang, 2019). Due to the lack of a boiling process, the pharmacological effects of the powdered formula YYFZ are simple a sum of the individual effects of its two component botanical drugs, Coicis semen and Fuzi. Hence, it is advisable to separately review the pharmacological effects and cardiovascular protective attributes of Coicis semen and Fuzi to understand the YYFZ’s pharmacological effects.

### 2.1 Coicis semen

Coicis semen, also known as Coix seed (*Coix lacryma-jobi* L. var. *ma-yuen* Stapf), is a TCM widely recognized for its rich nutritional and medicinal value. Fingerprinting of Coicis semen from dried tissues using the sequence-independent microarray (Niu et al., 2013) and the high-performance liquid chromatography (HPLC)-evaporative light scattering detector (Du et al., 2021) has been reported. It contains more than 80 active components including polysaccharides, fatty acids (FAs), phenolic acids, sterols, lipids, flavonoids, triterpenes, alkaloids, and adenosine (Zhu, 2017; Yu J. et al., 2022; Huang et al., 2022; Pan et al., 2023; Wei et al., 2023). The HPLC-atmospheric pressure chemical ionization-mass spectrometry (MS) method identified 12 triacylglycerols in coix oil (Xiang et al., 2005). Among them, the contents exceeding 3% include glycerol trioleate, glycerol dioleate, glycerol palmitate, and glycerol linoleate, comprising more than 90% of the triglycerides. The chromatographic fingerprint revealed the main pharmacologically active ingredients of Coicis semen, including 1,3-Dioleoyl-2-palmitoylglycerol, 1,2-dilinoleoyl-3-oleoyl-rac-glycerol, 1,3-Dipalmitoyl-2-Linolein, 1,2-Dilinoleoyl-3-palmitoyl-rac-glycerol, 1,2-Dioleoyl-3-linoleoyl-rac-glycerol, and glycerol trioleate (Du et al., 2021). These components are primarily responsible for improving lipid metabolism, antioxidation, and anti-inflammation, providing analgesic effects, and offering cardiovascular protection (Zhu R. et al., 2020; Chen et al., 2022; Yang et al., 2022d; Zeng et al., 2022; Pan et al., 2023), thereby supporting proper heart function and protecting against CVD (Xu et al., 2017; Chen et al., 2022). Studies have shown that Coicis semen has positive effects on CHDs (Yu F. et al., 2011; Wang et al., 2012), atherosclerosis (Check and K'Ombut, 1995; Yu F. et al., 2011), myocardial ischemia, MI(Yu J. et al., 2022), hypertension (Li et al., 2017; Chen et al., 2020), hypercholesterolemia (Wang et al., 2012), and hyperlipidemia (Chen et al., 2022).

### 2.2 Fuzi

Fuzi, also known as Aconiti Lateralis Radix Praeparata (*Aconitum carmichaelii* Debx.), is another TCM that contains more than 120 chemical components. HPLC-MS fingerprinting and Quadrupole Time-of-Flight (Q-TOF)-MS were used to analyze the chemical ingredients of Fuzi decoction sediments. As a result, 28 compounds were identified, with 25 of their structures confirmed (Zhang et al., 2012). The main components found in Fuzi include flavonoids, alkaloids such as aconitine (AC), salsolinol (SAL), fuziline (FZL), mesaconitine (MA), higenamine (HG), and hypaconitine (HA) (Zhao et al., 2012; Zhang et al., 2022d), polysaccharides, flavonoids, FAs, and ceramides (Zhang et al., 2022d; Zhang W. et al., 2023). Zhang et al. employed chromatographic fingerprinting to quantitatively analyze the diterpenoid alkaloids (DAs) of Fuzi, and utilized HPLC coupled with hybrid ion trap-time-of-flight mass spectrometry for qualitative assessment (Zhang et al., 2016). Among the samples, HA was the most abundant alkaloid in Fuzi, with a content range of 325.25–835.80 μg/g, followed by MA (96.41–541.62 μg/g), benzoylmesaconine (83.47–313.79 μg/g), AC (31.94–139.45 μg/g), benzoylhypaconine (22.39–96.93 μg/g), and benzoylaconine (BAC) (9.76–79.72 μg/g). In total, 99 DAs were identified and characterized, including 9 with clear identities, 77 with probable assignments, and 13 unknown compounds. However, HPLC fingerprinting demonstrated that the alkaloid content of Fuzi varies by geographical origin (Luo et al., 2019; Miao et al., 2019; Wang X. Y. et al., 2020; Ding et al., 2022; Qu et al., 2023). Luo et al. utilized HPLC coupled with an evaporative light-scattering detector to quantify the alkaloids. They observed average alkaloid contents in Fuzi as follows: HA at 1168.25 μg/g, MA at 1133.80 μg/g, Neoline at 766.62 μg/g, FZL at 750.32 μg/g, Songorine at 451. 89 μg/g, AC at 259.95 μg/g, and benzoylmeaconitine at 158.18 μg/g (Luo et al., 2019). Components of Fuzi, particularly alkaloids and polysaccharides, contribute to its antioxidant activities, anti-inflammatory properties, regulation of apoptosis, analgesic properties, and cardiotonic effects (Zhao et al., 2012; Yan et al., 2020; Yang et al., 2021b; Zhang et al., 2022d; Tian et al., 2022; Hacanli et al., 2023). However, HPLC-Q-TOF-MS and ultra-HPLC fingerprinting revealed distinct main activities among the different processed products of Fuzi (Zheng et al., 2014; Tong et al., 2021). In total, Fuzi helps protect against various CVD, particularly HF, and supports overall cardiovascular health (Yang et al., 2019; Tai et al., 2022). Long-term use of Fuzi is beneficial in preventing cardiovascular events (Tai et al., 2022).

## 3 The impact of YYFZ on mitochondria-ER interactions through data mining

Data-mining research has demonstrated that YYFZ can affect mitochondria-ER interactions by regulating mitochondrial function and ER function (Wang, 2019; Wang Y. et al., 2023; Wang Y. Y. et al., 2023). In addition, network pharmacology has shown that YYFZ can modulate the phosphoinositide 3-kinase (PI3K)/AKT, modulating mitogen-activated protein kinase (MAPK), and Ras signaling pathways (Wang Y. et al., 2023), all of which potentially affect mitochondria-ER interactions. Abnormal mitochondrial-ER interactions are thought to be key mechanisms in CVD such as CHD, HF, pulmonary hypertension, and atherosclerosis (Zhang et al., 2020). It can be speculated that the cardiovascular protective effect of YYFZ may be related to maintaining the homeostasis of mitochondria-ER interactions.

Studies have shown that the YYFZ may intervene in CHF through MAPK3(Wang Y. Y. et al., 2023). MAPK3 belongs to the MAPK family, which participates in diverse cellular functions such as inflammation, apoptosis, proliferation, and differentiation. Both in the *in-vivo* and *in-vitro* experiments, upregulation of MAPK3 attenuates ischemia/reperfusion (I/R) injury by reducing cardiomyocyte apoptosis (Wang H. et al., 2021). However, inhibiting MAPK phosphorylation levels in CHF model rats activates the peroxisome proliferator-activated receptor γ (PPAR-γ) pathway by participating in mitochondrial biogenesis, thereby promoting energy generation, improving cardiac energy metabolism, and slowing the pathological progression of CHF (Wang Y. W. et al., 2020).

Through metabolomics and network pharmacology analysis, it was found that YYFZ improves the efficiency of endoplasmic reticulum-associated protein degradation (ERAD), a process that removes misfolded proteins from the ER. By enhancing ERAD, it can reduce the accumulation of misfolded proteins and subsequently alleviate ER stress (Wang Y. et al., 2023). ER stress is a cellular condition that occurs when there is an accumulation of unfolded or misfolded proteins in the ER (Haeri and Knox, 2012), which triggers a cellular response known as the UPR (Hetz, 2012). It is speculated that YYFZ may also have the ability to modulate the UPR, therefore helping restore ER homeostasis and reducing the burden on the ER. Another clinical metabolomics combined with network pharmacology analysis showed that HG, β-sitosterol, and stigmasterol in YYFZ can bind to ER proteins and affect the sphingomyelin pathway, thereby reducing intravascular lipid accumulation, inhibiting inflammation, and exerting potential therapeutic effects on the cardiovascular system (Wang Y. et al., 2022). The study also suggests that ASM enzymes, a part of the sphingomyelin pathway, may be key targets for the treatment of CHD.

## 4 The potential impact of YYFZ on mitochondria-ER interactions

A mouse study found that YYFZ enhanced the biological activity of nitric oxide (NO), restored mitochondrial membrane potential (MMP), and reduced reactive oxygen species (ROS) generation, thereby alleviating mitochondrial and vascular endothelial dysfunction and improving the pathological state of myocardial ischemia (Li et al., 2023). However, direct laboratory evidence demonstrating YYFZ’s effects on mitochondria-ER interactions is still lacking. Further exploration is needed to elucidate the potential mechanism. Nevertheless, the constituent botanical drugs of YYFZ, Coicis semen and Fuzi, have been shown to play a significant role in regulating mitochondria-ER interactions and conferring cardiovascular protection individually.

### 4.1 Regulating cardiovascular risk factors

#### 4.1.1 Regulating lipid metabolism

**FIGURE 2:**
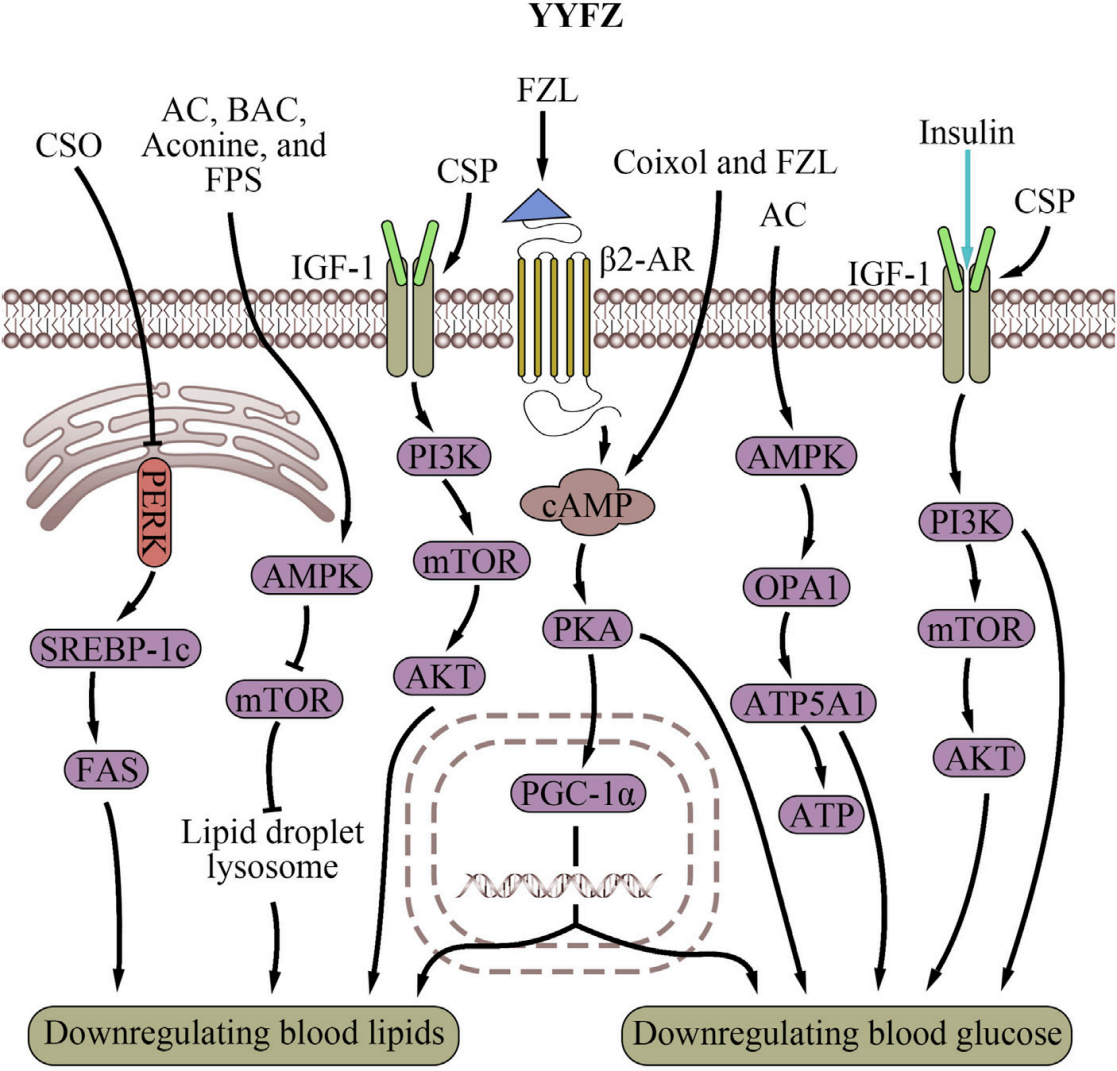
Cardiovascular risk factor regulatory effects of YiyiFuzi Powder (Abbreviations: AC, aconitine; AMPK, AMP-activated protein kinase; ATP, adenosine triphosphate; ATP5A1, adenosine triphosphate synthase, H+ transporting, mitochondrial F1 complex, alpha subunit 1; BAC, benzoylaconine; β2-AR, β2-adrenergic receptor; cAMP, cyclic adenosine monophosphate; CSO, Coix seed oil; CSP, Coix seed polysaccharides; FAS, fatty acid synthase; FPS, polysaccharides from Fuzi; FZL, fuziline; IGF-1, insulin-like Growth Factor 1; mTOR, mechanistic target of rapamycin; OPA1, optic atrophy 1; PERK, protein kinase RNA-like endoplasmic reticulum kinase; PGC-1α, peroxisome proliferator-activated receptor-γ coactivator-1α; PI3K, phosphoinositide 3-kinase; PKA, protein kinase A; SREBP-1c, sterol regulatory element-binding protein-1c; YYFZ, YiyiFuzi Powder).

YYFZ potentially modulates mitochondria-ER interactions to regulate cardiometabolic risk factors such as lipids and glucose (Table 1; Figure 2), thereby exerting a cardiovascular protective effect. Dysregulated lipid metabolism is a major risk factor for CVD (Zeng et al., 2022). Coicis semen has shown effects on lipid metabolism by inhibiting fatty acid synthase (FAS), decreasing cholesterol synthesis, and supporting immune functions (Chen et al., 2022; Zhao et al., 2023). Studies found that Coicis semen decreases serum total cholesterol (TC), low-density lipoprotein cholesterol (LDL-C), and triglycerides (TG) in animal models (Lin and Tsai, 2008; Wang et al., 2012; Zhang et al., 2022c; Yang et al., 2022d). Coicis semen is rich in branched-chain amino acids (BCAAs), especially leucine (Liu et al., 2015). In mice, leucine decreased hepatic TG accumulation and atherosclerotic lesion area, exhibiting lipid-lowering and cardiovascular protective properties (Yokota et al., 2016; Zhao et al., 2016). Coix seed polysaccharides (CSP), active components of Coicis semen, have been found to alleviate type 2 diabetes mellitus in mouse models by increasing high-density lipoprotein cholesterol levels (Yu W. et al., 2022).

**TABLE 1:** Cardiovascular risk factor regulatory effects of YYFZ’s major components.

Herbal medicine	Components	Pathways/Targets	Potential effect
*Coix lacryma-jobi* L.var. *ma-yuen* Stapf	CSO	Potentially suppressing the PERK/SREBP-1c/FAS signaling pathway	Protecting mitochondrial function from lipid droplet-induced stress
	CSP	Activating the IGF-1/PI3K/AKT signaling pathway	Decreasing plasma triglyceride levels and regulating cholesterol levels
*Aconitum carmichaelii* Debx.	AC, BAC, Aconine, and FPS	Activating the AMPK signaling pathway	Improving energy metabolism, regulating lipid, and mitochondrial biogenesis
	FZL	Activating β-ARs thereby activating the cAMP/PKA signaling pathway	Enhancing mitochondrial energy metabolism and stimulating liver lipolysis
*Coix lacryma-jobi* L.var. *ma-yuen* Stapf	CSP	Activating the IGF-1/PI3K/AKT signaling pathway	Maintaining normal insulin sensitivity and increasing glucose uptake and utilization
	Coixol	Activating the cAMP/PKA pathway thereby increasing PGC-1α expression	Enhancing glucose-stimulated insulin secretion, and resulting in more mitochondria formed in cells
*Aconitum carmichaelii* Debx.	FZL	Activating the cAMP/PKA signaling pathway	Enhancing mitochondrial energy metabolism and increasing liver glycogenolysis
	AC	Activating the AMPK/OPA1/ATP5A1 signaling pathway	Mediating mitochondrial function and impacting glucose handling

AC, aconitine; AMPK, AMP-activated protein kinase; ATP5A1, ATP, synthase, H+ transporting, mitochondrial F1 complex, alpha subunit 1; BAC, benzoylaconine; β-AR, β-adrenergic receptor; cAMP, cyclic adenosine monophosphate; CSO, coix seed oil; CSP, coix seed polysaccharides; FAS, fatty acid synthase; FPS, polysaccharides from Fuzi; FZL, fuziline; IGF-1, insulin-like Growth Factor 1; OPA1, optic atrophy 1; PERK, protein kinase RNA-like endoplasmic reticulum kinase; PGC-1α, peroxisome proliferator-activated receptor-γ coactivator-1α; PI3K, phosphoinositide 3-kinase; PKA, protein kinase A; SREBP-1c, sterol regulatory element-binding protein-1c; YYFZ, YiyiFuzi Powder.

In obese mice, Coix seed oil (CSO) protects mitochondrial function from lipid droplet-induced stress (Chen et al., 2022). Obesity increases oxidative stress, inflammation, and ER stress via the protein kinase RNA-like endoplasmic reticulum kinase (PERK)/sterol regulatory element-binding protein-1c (SREBP-1c)/FAS signaling pathway (Xue et al., 2005; Zhang and Kaufman, 2008; Han and Kaufman, 2016; Saltiel and Olefsky, 2017; Wang S. et al., 2021). PERK is an ER-resident transmembrane kinase that is a part of the UPR. PERK is activated when the ER is under stress, leading to the activation of SREBP-1c, which promotes lipogenesis (Han and Kaufman, 2016). CSO may exert cardiovascular protection by reducing oxidative and ER stress and protecting mitochondria through suppressing the PERK/SREBP-1c/FAS signaling pathway. Another study found that Coix seed extracts (CSEs) reduced chronic inflammation, insulin resistance, weight gain, fat content, and lipid levels in obese mice (Zhang et al., 2022c). Researchers speculated that this reduction was due to the CSE group’s reduction of liver fat weight and lipid control as a result of reduced ER stress.

Active components in Fuzi also contribute to YYFZ’s lipid-regulating effects. Studies show that Fuzi can lower LDL-C and TC, preventing atherosclerosis (Huang et al., 2010). The potential mechanism is that Fuzi upregulates the expression of fatty-acid-metabolism genes such as Acot1, Acot2, Acsl3, Acox3, and Acox2, which are involved in FA hydrolysis, beta-oxidation, and acyl-CoA production (Yu et al., 2012). Additionally, Fuzi was identified as improving energy metabolism, including lipid regulation, through the crucial AMP-activated protein kinase (AMPK) signaling pathway (Deng et al., 2019). This pathway ensures energy homeostasis and mitochondrial biogenesis (Hardie and Sakamoto, 2006; Jager et al., 2007). The diterpene alkaloid in Fuzi, FZL (Xiong et al., 2012; Fan et al., 2020), activates β-adrenergic receptors (β-AR), which then activate the downstream cyclic adenosine monophosphate (cAMP)/protein kinase A (PKA) signaling pathway. This enhances mitochondrial energy metabolism and stimulates liver lipolysis to improve lipid profiles (Gao et al., 2023). Overall, YYFZ helps maintain mitochondrial function, biogenesis, energy metabolism, and ER stress through its upregulation of the cAMP/PKA signaling pathways, and downregulation of the PERK/SREBP-1c/FAS signaling pathways, thereby modulating lipid metabolism and protecting the cardiovascular system.

#### 4.1.2 Regulating blood glucose metabolism

Regulating blood glucose is another important way that YYFZ protects cardiovascular health. CSP, Coixol, FZL, and AC are the main constituents contributing to the glucose regulation effects of YYFZ. CSP increased serum insulin levels to alleviate type 2 diabetes mellitus in mouse models (Yu W. et al., 2022). CSP treatment also modulated gut microbiota composition via activation of the insulin-like Growth Factor 1 (IGF-1)/PI3K/AKT signaling pathway (Xia et al., 2021). The IGF-1/PI3K/AKT signaling pathway plays a significant role in regulating blood glucose, primarily involving maintaining normal insulin sensitivity, increasing glucose uptake and utilization, decreasing plasma triglyceride levels, and regulating cholesterol levels (Clemmons, 2004; Ng et al., 2008; Jensen-Cody and Potthoff, 2021). Additionally, this pathway is closely related to the structure and function of mitochondria (Radovic et al., 2019) and ER (Novosyadlyy et al., 2008). The IGF-1 signaling pathway supports mitochondrial biogenesis and function by maintaining MMP, increasing ATP production, reducing mitochondrial ROS production, and promoting mitochondrial protein complexes (Puche et al., 2008). IGF-1 also interacts with the ER (Paharkova-Vatchkova and Lee, 2010). Additionally, PI3K signaling regulates mitochondrial physiology at MAM through mTORC2 and AKT action, controlling mitochondrial metabolism, membrane integrity, and cell survival (Betz et al., 2013). The IGF-1/PI3K/AKT signaling pathway also regulates Ca^2+^ exchange between the ER and mitochondria (Lv Y. et al., 2022).

Coixol, an alkaloid extracted from Coicis semen, enhances glucose-stimulated insulin secretion via the cAMP/PKA signaling pathway (Hameed et al., 2019; Wang et al., 2021d). This pathway may interact with peroxisome proliferator-activated receptor γ coactivator 1α (PGC-1α) to modulate mitochondrial biogenesis, an important regulator of blood sugar (Wang et al., 2021d). It is plausible that Coixol increases cAMP levels to activate PGC-1α, resulting in more mitochondria formed in cells, thereby playing a significant role in sugar consumption and regulating blood sugar levels (Kim et al., 2008; Kwak et al., 2010; Wang et al., 2021d).

FZL increases mitochondrial function in muscle, enhancing glucose transport and lowering blood glucose (Gao et al., 2023). Interestingly, this process is associated with the rise in mitochondrial temperatures and MMP. Additionally, by enhancing mitochondrial energy metabolism, FZL also increases liver glycogenolysis via the cAMP/PKA signaling pathway (Gao et al., 2023). AC, another alkaloid found in Fuzi, improves energy metabolism disorders, resulting in the remodeling of mitochondrial function and mediating mitochondrial function via upregulating the AMPK/optic atrophy 1 (OPA1)/adenosine triphosphate synthase, H+ transporting, mitochondrial F1 complex, alpha subunit 1 (ATP5A1) signaling pathway (Qiu et al., 2021). AMPK also stimulates glucose metabolism in the ER (Long and Zierath, 2006). By activating AMPK, Fuzi may impact glucose handling and reverse diabetic cardiomyopathy (DCM), suppressing high glucose-induced effects on MAM formation, mitochondrial Ca^2+^ increase, and mitochondrial dysfunction (Wu S. et al., 2019). In summary, both botanical drugs in YYFZ likely work in coordination to regulate blood sugar levels through modulating the IGF-1/PI3K/AKT, cAMP/PKA, and AMPK/OPA1/ATP5A1 pathways which are involved in mitochondrial biogenesis, function, and interactions with the ER. This helps protect cardiovascular health by mitigating hyperglycemia-related risk.

### 4.2 Impact of YYFZ on mitochondria

#### 4.2.1 Regulating mitochondrial biogenesis and structure

Mitochondrial biogenesis is the process of generating new mitochondria within cells. BCAAs in Coicis semen along with intermediate metabolites such as C3 and C5 acylcarnitine, acetyl-CoA, succinyl-CoA, and 3-hydroxyisobutyrate, directly induce mitochondrial biogenesis through protein acetylation and phosphorylation modifications, transcriptional regulation by promoting the expression of mitochondrial transcription factor A mRNA, and increasing mtDNA copy number (Ye et al., 2020). PGC-1α is another underlying pharmacological mechanism of BCAAs generating mitochondria, extensively described as a master regulator of mitochondrial biogenesis in skin and muscle tissues (Mootha et al., 2003; Nisoli et al., 2003). BCAAs promote the gene expression of PGC-1α through the endothelial nitric oxide synthase/NO/cGMP/sirtuin 1 (SIRT1) and SIRT1/Liver kinase B1 (LKB1)/AMPK signaling pathway (D'Antona et al., 2010; Hatazawa et al., 2014; Liang et al., 2014; Schnuck et al., 2016; Chen et al., 2019). Furthermore, leucine also directly activates SIRT1 and the mammalian target of rapamycin (mTOR) to promote PGC-1α expression (Liang et al., 2014; Schnuck et al., 2016; Chen et al., 2019).

**FIGURE 3:**
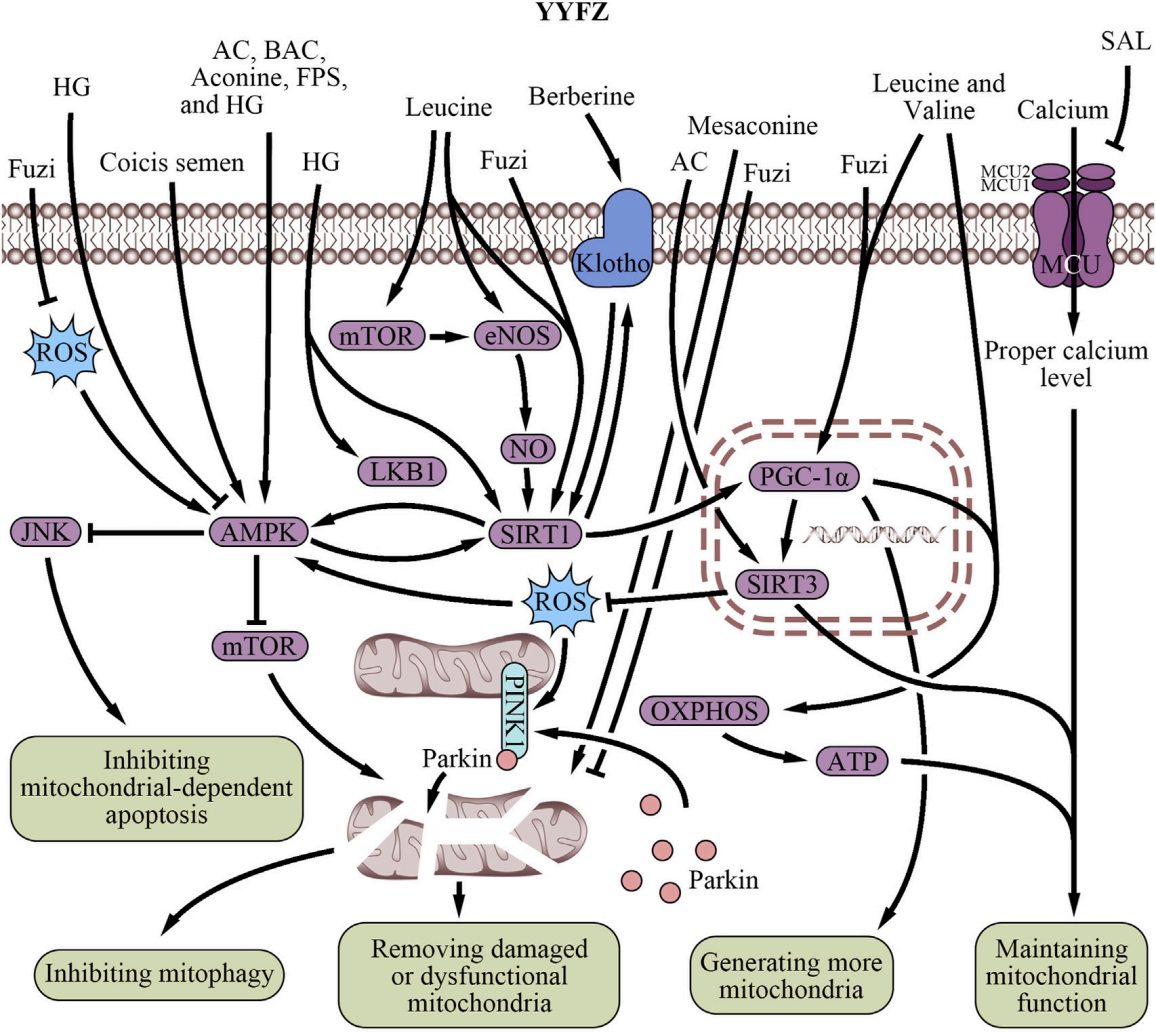
Regulatory effects of YiyiFuzi Powder’s major components on mitochondrial structure and function (Abbreviations: AC, aconitine; AMPK, AMP-activated protein kinase; ATP, adenosine triphosphate; BAC, benzoylaconine; eNOS, endothelial nitric oxide synthase; FPS, polysaccharides from Fuzi; HG, higenamine; JNK, c-Jun N-terminal kinase; LKB1, liver kinase B1; MCU, mitochondrial calcium uniporter; mTOR, mechanistic target of rapamycin; NO, nitric oxide; OXPHOS, oxidative phosphorylation; PGC-1α, peroxisome proliferator-activated receptor-γ coactivator-1α; PINK1, PTEN-induced putative kinase 1; ROS, reactive oxygen species; SAL, salsolinol; SIRT, sirtuin; YYFZ, YiyiFuzi Powder).

Several active compounds in Fuzi, such as AC, BAC, and aconine, significantly increase mitochondrial mass (Park et al., 2016) by activating the AMPK signaling pathway (Wu J. et al., 2016; Deng et al., 2019), which induces mitochondrial biogenesis by regulating morphology and suppressing ROS-associated ER stress (Wu and Zou, 2020). Additionally, Fuzi formulations like Qili Qiangxin Capsule (QLQX) enhance cardiomyocyte metabolism, mitochondrial content, and mitochondrial biogenesis via PGC-1α activation (Lin et al., 2015). Fuzi also increases heart mitochondrial biogenesis when combined with Zingiberis Rhizoma (ZR), potentially via the SIRT1/PGC-1α signaling pathway (Lu et al., 2017). YYFZ botanical drugs support mitochondrial biogenesis (Table 2; Figure 3) by coordinating the effects of Coicis semen and Fuzi on mitochondrial quantity, quality, and gene expression networks.

**TABLE 2:** Regulatory effects of YYFZ’s major components on mitochondrial structure and function.

Herbal medicine	Components	Pathways/Targets	Potential effect
*Coix lacryma-jobi* L.var. *ma-yuen* Stapf	Leucine	Activating the eNOS/NO/cGMP/SIRT1 and the SIRT1/LKB1/AMPK signaling pathways, thereby promoting PGC-1α expression	Generating mitochondria
	Leucine	Activating SIRT1 and mTOR thereby promoting PGC-1α expression	Generating mitochondria
*Aconitum carmichaelii* Debx.	AC, BAC, and Aconine	Activating the AMPK signaling pathway	Inducing mitochondrial biogenesis
	—	Activating PGC-1α expression	Enhancing mitochondrial content and mitochondrial biogenesis
	—	Activating the SIRT1/PGC-1α signaling pathway	Increasing heart mitochondrial biogenesis
*Coix lacryma-jobi* L.var. *ma-yuen* Stapf	Berberine	Increasing the Klotho/SIRT1 signaling pathway	Alleviating mitochondrial dysfunction
	Leucine and valine	Enhancing the mitochondrial OXPHOS/ATP signaling pathway	Preventing decline in mitochondrial respiration, mitochondrial damage, and reduction in ATP production
	Leucine and valine	Upregulating PGC-1α expression	Generating mitochondria
*Aconitum carmichaelii* Debx.	SAL	Inhibiting the MCU signaling pathway	Improving mitochondrial respiratory function and energy metabolism
	Mesaconine	Activating the PINK1-dependent mitophagy signaling pathway	Removing damaged or dysfunctional mitochondria to maintain mitochondrial population health and function
	FPS	—	Attenuating MMP
	AC	Activating SIRT3 activity	Inhibiting the opening of the mPTP, and improving mitochondrial energy metabolism
	HG	Upregulating LKB1, AMPKα1, and SIRT1 expression	Alleviating disturbances in myocardial mitochondrial energy metabolism
	—	Activating the PPARα/PGC-1α/SIRT3 signaling pathway	Promoting mitochondrial energy metabolism
	—	Suppressing the ROS/AMPK/mTOR signaling pathway	Removing damaged mitochondria from cells
	—	Suppressing the PINK1/Parkin-mediated mitophagy signaling pathway	Removing damaged mitochondria from cells
		Increasing Cu-Zn SOD level and Mn-SOD level, while decreasing MDA level	Protecting mitochondrial membrane integrity

AC, aconitine; AMPK, AMP-activated protein kinase; ATP, adenosine triphosphate; BAC, benzoylaconine; HG, higenamine; cGMP, cyclic guanosine monophosphate; Cu-Zn SOD, copper-zinc superoxide dismutase; eNOS, endothelial nitric oxide synthase; FPS, polysaccharides from Fuzi; LKB1, liver kinase B1; MCU, mitochondrial calcium uniporter; MDA, malondialdehyde; MMP, mitochondrial membrane potential; Mn-SOD, manganese superoxide dismutase; mPTP, mitochondrial permeability transition pore; mTOR, mechanistic target of rapamycin; NO, nitric oxide; OXPHOS, oxidative phosphorylation; PGC-1α, peroxisome proliferator-activated receptor-γ coactivator-1α; PINK1, PTEN-induced putative kinase 1; PPARα, peroxisome proliferator-activated receptor α; ROS, reactive oxygen species; SAL, salsolinol; SIRT, sirtuin; YYFZ, YiyiFuzi Powder; 3-HIB, 3-hydroxyisobutyrate.

#### 4.2.2 Regulating mitochondrial function and energy metabolism

Mitochondrial dysfunction plays a significant role in ischemic CVD caused by endothelial dysfunction (Song et al., 2013). Most compounds from Coicis semen primarily impact mitochondrial function rather than mitochondrial biogenesis or structure (Table 2; Figure 3). Berberine, a bioactive component of Coicis semen, may increase the Klotho/SIRT1 signaling pathway to alleviate mitochondrial dysfunction and cardiac senescence (Zhang T. et al., 2023). Klotho is an anti-aging protein that effectively reduces oxidative stress, inhibits apoptosis, and preserves mitochondrial function (Li et al., 2022; Donate-Correa et al., 2023). This protective effect was found in doxorubicin (DOX)-induced senescent H9c2 cells and naturally aged rats (Li et al., 2022).

Energy metabolism plays a crucial role in mitochondrial function. In recent years, the relationship between mitochondria and cardiomyocyte energy metabolism has gradually become a hot topic in clinical research. Efficient mitochondrial respiration is essential for ATP synthesis which preserves the energy supply (Aon et al., 2015; Viloria et al., 2022), reduces oxidative stress (Tsutsui et al., 2009; Kowalczyk et al., 2021), and prevents heart damage (Powers et al., 2014). CSEs have been shown to reduce the consumption of reducing sugars by the probiotic bacterium *L. reuter*i while increasing its growth (Yang et al., 2022c). This indicates that CSEs might be involved in energy supply and mitochondrial function. CSO enhances mitochondrial respiration and ATP production, thereby regulating blood sugar and improving overall energy metabolism (Chen et al., 2022). BCAAs like leucine and valine in Coicis semen are linked to lower CVD risk (Zheng et al., 2022), and they synergize with FAs to upregulate PGC-1α expression and enhance the mitochondrial oxidative phosphorylation (OXPHOS)/ATP signaling pathway (Ye et al., 2020). In coronary artery disease patients, a shift in mitochondrial metabolism from OXPHOS to glycolysis was observed (Ait-Aissa et al., 2019). The reduced OXPHOS leads to decreased mitochondrial respiration, mitochondrial damage, decreased ATP production, and a corresponding rise in glycolytic flux, all of which can contribute to the progression of heart disease (Ait-Aissa et al., 2019). Thus, BCAAs could help prevent this shift and mitochondrial damage by enhancing OXPHOS to support cardiovascular health.

Fuzi can be used in the treatment of CVD especially in HF, due to its valuable cardiotonic effect which helps improve heart function and enhance the contractility of the heart muscle (Zhao et al., 2012; Zhang J. et al., 2022). In addition, Fuzi has been studied for its potential anti-MI effects, including mitigating mitochondrial dysfunction caused by MI and restoring energy metabolism in the heart (Wu H. et al., 2019). This effect maintains mitochondrial health and function (Table 2; Figure 3), improves blood flow and oxygen delivery to the body’s tissues, and ensures an adequate supply of energy for the heart (Wang N. N. et al., 2021; Liu X. et al., 2022). SAL, a bioactive component of Fuzi, has been identified as a cardiotonic component (Zhou, 2011). Studies have shown that SAL improves mitochondrial function in H9c2 cardiomyocytes and attenuates DOX-induced CHF in rats (Wen et al., 2019b). This plant-based isoquinoline alkaloid ameliorates DOX-induced mitochondrial dysfunction and increases mitochondrial oxygen consumption rate (OCR) in H9c2 cardiomyocytes. It was suggested that SAL might improve mitochondrial respiratory function and energy metabolism by inhibiting excessive activation of the mitochondrial calcium uniporter (MCU) pathway in H9c2 cells (Wen et al., 2019b). Mesaconine, another bioactive component of Fuzi, activates the cardiac phosphatase and tensin homolog (PTEN)-induced putative kinase 1 (PINK1)-dependent mitophagy pathway (Zhou et al., 2023), which also helps remove damaged or dysfunctional mitochondria to maintain overall mitochondrial population health and function (Wallace et al., 2020; Zhou et al., 2023). Besides, AC activates SIRT3 activity in mitochondria, reducing the degree of Cyp-D acetylation, inhibiting the opening of the mitochondrial permeability transition pore, and improving mitochondrial energy metabolism (Wang et al., 2019). These biological processes protect myocardial tissue. Furthermore, water-soluble alkaloids in Fuzi also modulate mitochondria-mediated pathways to promote mitochondrial energy metabolism (Zhang J. et al., 2022).

HG, a natural benzylisoquinoline alkaloid found in Fuzi (Zhang et al., 2022d), was reported to upregulate mRNA and protein expression, such as LKB1, AMPKα1, and SIRT1, alleviating disturbances in myocardial mitochondrial energy metabolism when combined with 6-gingerol (Wen et al., 2020). The combination of Fuzi and Zingiber officinale (ZR) has also been found to promote mitochondrial energy metabolism via activating the peroxisome proliferator-activated receptor α (PPARα)/PGC-1α/SIRT3 signaling pathway (Wen et al., 2019a; Wen et al., 2019b). This is particularly relevant to HF, as energy metabolism is often impaired in this condition (Xing et al., 2023). Several TCM formulas containing Fuzi and other botanical drugs have been shown to improve mitochondrial energy metabolism and function. QLQX has been shown to enhance metabolism in cardiomyocytes, reducing cardiomyocyte apoptosis and improving heart function in infarcted hearts via suppression of the ROS/AMPK/mTOR signaling pathway (Fan et al., 2022) and PINK1/Parkin-mediated mitophagy (Zhou et al., 2020). This involves removing damaged mitochondria from cells, helping maintain their function and protect against cell death. A study showed that Sini decoction reduced lipid peroxidation incidence in myocardial mitochondria by increasing levels of antioxidant enzymes (copper-zinc superoxide dismutase and manganese superoxide dismutase, Mn-SOD (Zhao et al., 2005)) and decreasing malondialdehyde level, further protecting mitochondrial membrane integrity and reducing HF incidence (Zhao et al., 2010). Yang-xin-xue Keli, a TCM formula containing Fuzi, was found to improve cardiac function and reverse myocardial loss and fibrosis in rats with DOX-induced HF. This effect was attributed to the regulation of mitochondrial homeostasis and function (Long et al., 2022).

#### 4.2.3 Regulating mitochondrial membrane potential

Regulating MMP is another critical aspect of regulating mitochondrial function (Table 2). The MMP is essential for various cellular processes, including ATP production, mitochondrial homeostasis, and the maintenance of mitochondrial quality (Vasan et al., 2022). Polysaccharides are beneficial to the cardiovascular system (Yin et al., 2018) and have been found to regulate MMP(Wang et al., 2014). Studies have identified that some polysaccharides exert a cardiovascular protective effect by maintaining MMP, increasing ATP generation, and increasing the OCR of cardiomyocytes (Zuo et al., 2018), which is crucial for energy production in cardiomyocytes. Both Coicis semen and Fuzi contain large amounts of polysaccharides, which are the main components of YYFZ that exert potential MMP regulation effects. Liao et al. demonstrated that polysaccharides from Fuzi (FPS) treatment attenuate starvation-induced cell viability reduction and MMP collapse in a concentration-dependent manner (Liao et al., 2013). Nevertheless, MMP collapse is a standard indicator of mitochondrial damage (Hussain et al., 2008). CSP and FPS may potentially protect mitochondria by regulating MMP collapse, thereby serving cardiovascular aims.

### 4.3 Regulating ER stress

**FIGURE 4:**
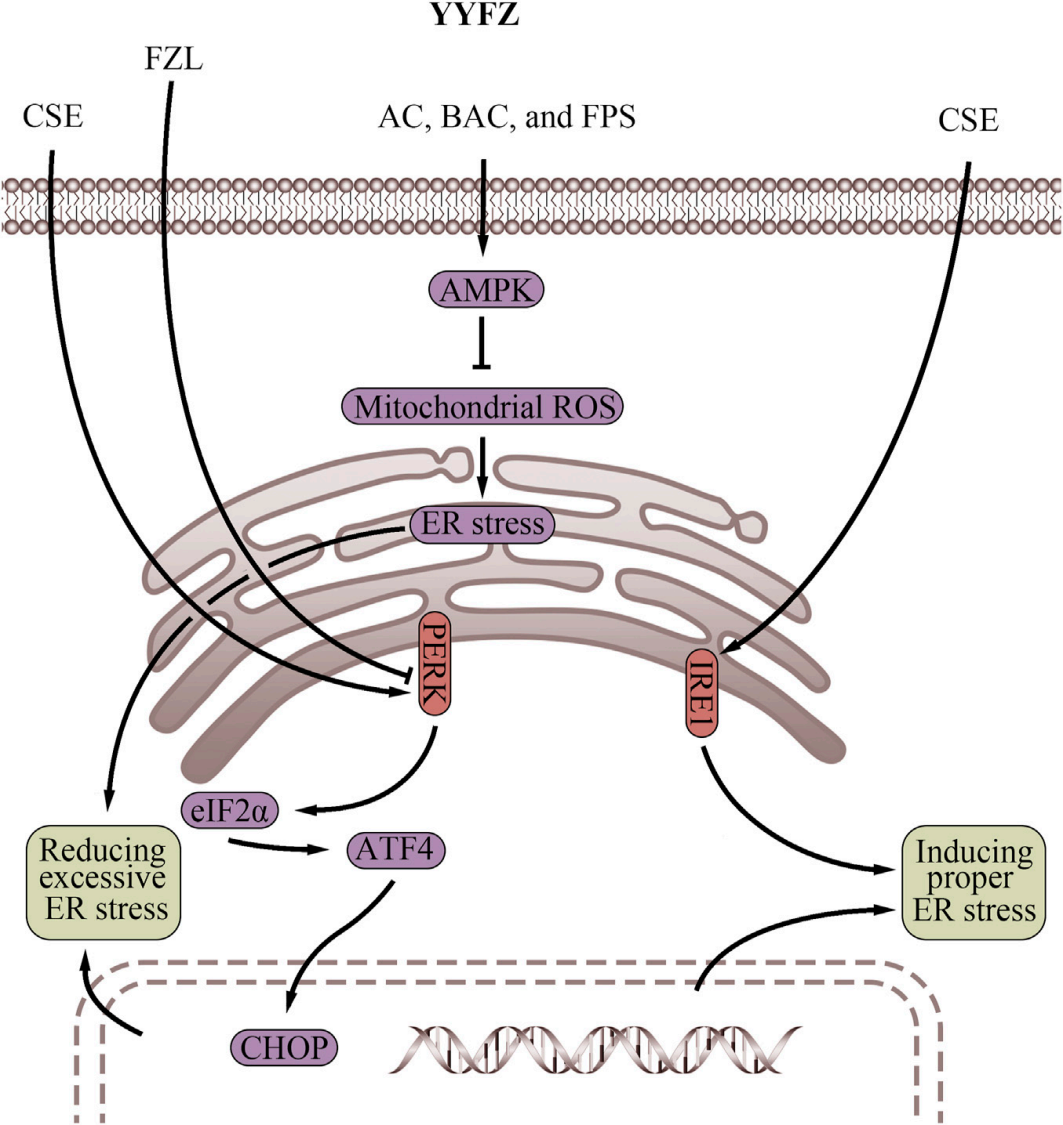
Endoplasmic reticulum regulatory effects of YiyiFuzi Powder (Abbreviations: AC, aconitine; AMPK, AMP-activated protein kinase; ATF4, activating transcription factor 4; BAC, benzoylaconine; CHOP, C/EBP homologous protein; CSE, Coix seed extract; eIF2α, eukaryotic initiation factor 2 alpha; ER, endoplasmic reticulum; FPS, polysaccharides from Fuzi; FZL, fuziline; IRE1, inositol-requiring enzyme 1; PERK, protein kinase RNA-like endoplasmic reticulum kinase; ROS, reactive oxygen species; YYFZ, YiyiFuzi Powder).

The ER stress response and the UPR, triggered by ER stress, play crucial roles in maintaining cardiomyocyte homeostasis (Table 3; Figure 4), which are important for cardiovascular health (Zhang et al., 2017; Ren et al., 2021; Pietrafesa et al., 2023). Excessive ER stress disrupts secretory pathways and contributes to the progression of CVD (Zhang et al., 2017; Fu and Doroudgar, 2022). However, proper ER stress and early UPR response during cell injury may play a protective role in the cardiovascular system by maintaining cellular homeostasis, reducing tissue damage, and facilitating tissue repair (Glembotski, 2008; Kitakaze and Tsukamoto, 2010; Wang et al., 2018). Therefore, strategies to modulate ER stress could benefit CVD prevention and treatment. Coicis semen has demonstrated beneficial effects on obesity and dyslipidemia, which may involve regulating ER stress. In Chang liver cells, an ethanol extract of Coicis semen induced ER stress and stimulated the UPR via activation of the PERK and IRE1 pathways (Kim et al., 2018). When subjected to ER stressors, cells react rapidly to halt their translational machinery via PERK activation (Wong et al., 1993; Harding et al., 1999). The PERK pathway is necessary to protect the heart from pressure overload-induced congestive HF(Liu et al., 2014). Mice with cardiac-specific PERK knockout exhibited impaired cardiac function and increased damage, such as left ventricular fibrosis, enhanced cardiomyocyte apoptosis, and exacerbated lung remodeling, in response to chronic pressure overload (Liu et al., 2014). Likewise, IRE1 activation rapidly occurs during ER stress and protects against myocardial I/R injury (DuRose et al., 2006; Wu T. et al., 2019). The IRE1 pathway also prevents cell death by inhibiting Bax- and Bak-mediated ER membrane permeabilization in CVD, which prevents the leakage of ER contents, accumulation of Ca^2+^ in the mitochondria, oxidative stress, and ultimately cell death (Siwecka et al., 2021). In summary, Coicis semen shows the potential to enhance ER stress, and thus activation of the PERK and IRE1 pathways is associated with cardiovascular protective effects (Liu et al., 2014; Wu T. et al., 2019). However, further research is warranted to fully elucidate the mechanisms and determine optimal dosing for Coicis semen to properly regulate ER stress in CVD treatment.

**TABLE 3:** Endoplasmic reticulum regulatory effects of YYFZ’s major components.

Herbal medicine	Components	Pathways/Targets	Potential effect
*Coix lacryma-jobi* L.var. *ma-yuen* Stapf	Ethanol CSE	Activating the PERK pathway	Inducing proper ER stress
	Ethanol CSE	Activating the IRE1 pathway thereby inhibiting Bax- and Bak-mediated ER membrane permeabilization	Inducing proper ER stress while preventing the leakage of ER contents, accumulation of calcium in the mitochondria, oxidative stress, and ultimately cell death
*Aconitum carmichaelii* Debx.	FZL	Downregulating the PERK/eIF2α/ATF4/CHOP pathway	Suppressing the excessive ER stress and reducing ER stress-induced apoptosis
	AC, BAC, and FPS	Activating the AMPK signaling pathway	Suppressing mitochondrial ROS-associated ER stress

AC, aconitine; AMPK, AMP-activated protein kinase; ATF4, activating transcription factor 4; BAC, benzoylaconine; CHOP, C/EBP, homologous protein; CSE, coix seed extract; eIF2α, eukaryotic initiation factor 2 alpha; ER, endoplasmic reticulum; FPS, polysaccharides from Fuzi; FZL, fuziline; IRE1, inositol-requiring enzyme 1; PERK, protein kinase RNA-like endoplasmic reticulum kinase; ROS, reactive oxygen species; YYFZ, YiyiFuzi Powder.

Both mitochondrial energy metabolism and apoptosis can be influenced by ER function which Fuzi has been shown to improve (Wu H. et al., 2019; Zhang J. et al., 2022). FZL was found to protect against isoproterenol (ISO)-induced myocardial injury *in vivo* by suppressing ER stress via downregulating the PERK/eukaryotic initiation factor 2 alpha (eIF2α)/activating transcription factor 4 (ATF4)/C/EBP homologous protein (CHOP) pathway (Fan et al., 2020). Western blot analysis further demonstrated that FZL reduced ER stress-induced apoptosis and effectively improved cardiac function in rats with ISO-induced myocardial injury (Fan et al., 2020). BAC, AC, and FPS have also been shown to activate the AMPK signaling pathway (Liao et al., 2013; Deng et al., 2019; Wang W. et al., 2022). Interestingly, activation of AMPK suppresses mitochondrial ROS-associated ER stress. Therefore, compounds in Fuzi may exert cardiovascular protection through AMPK activation, which serves to dampen mitochondrial ROS generation and subsequent ER stress induction (Wu and Zou, 2020). By alleviating aberrant mitochondria-ER crosstalk involved in CVD pathogenesis, the Fuzi components may help reduce mitochondrial dysfunction and protein misfolding in the ER. Further research is warranted to validate if AMPK stimulation represents a key mechanism through which Fuzi contributes to the cardiovascular effects of YYFZ. Properly controlling ER function through modulation of ER stress response pathways like PERK, IRE1, and AMPK could offer new opportunities for managing CVD. Further studies on YYFZ may provide valuable insights into developing improved strategies targeting this mechanism and fully elucidating potential drug interactions or side effects.

### 4.4 Reducing oxidative stress

The mitochondrial respiratory chain is the main inducer of oxidative stress by producing ROS(Petrosillo et al., 2003; Batandier et al., 2004; Paradies et al., 2004). Increased mitochondrial permeability can lead to elevated ROS production. This may contribute to the loss of myocardial contractility and increased myocardial stiffness, promoting cardiac remodeling processes (Gomez et al., 2007). Dysregulated ROS production and oxidative stress have been linked to cardiac hypertrophy, HF, cardiac I/R injury, and DCM (Peoples et al., 2019). A double-blind clinical trial using drugs targeting mitochondrial ROS may improve or even restore myocardial energetics, showing promise in limiting HF progression (Daubert et al., 2017). Amino acid metabolomics analysis has demonstrated that YYFZ regulates the glutathione and taurine synthesis pathways as well as phenylalanine and tyrosine metabolism (Wang Y. et al., 2023). Both glutathione and taurine are powerful antioxidant molecules that help protect cells from oxidative damage (Kerksick and Willoughby, 2005; Schaffer and Kim, 2018; Kwon et al., 2019; Surai et al., 2021), thereby safeguarding mitochondria from harm. YYFZ powder may enhance the synthesis of glutathione and taurine by regulating the metabolism of amino acids such as cysteine, glutamate, glycine, and methionine. Furthermore, phenylalanine and tyrosine serve as precursors for the synthesis of various antioxidant molecules (Szewczyk et al., 2021), including catecholamines and melanin. Metabolomic and network pharmacology analyses have also shown that YYFZ indirectly contributes to antioxidant effects by reducing lipid accumulation, increasing glucose utilization and ATP production, promoting the production of intermediates, donating electrons to the electron transport chain (ETC.), and driving ATP synthesis (Wang Y. et al., 2023).

**FIGURE 5:**
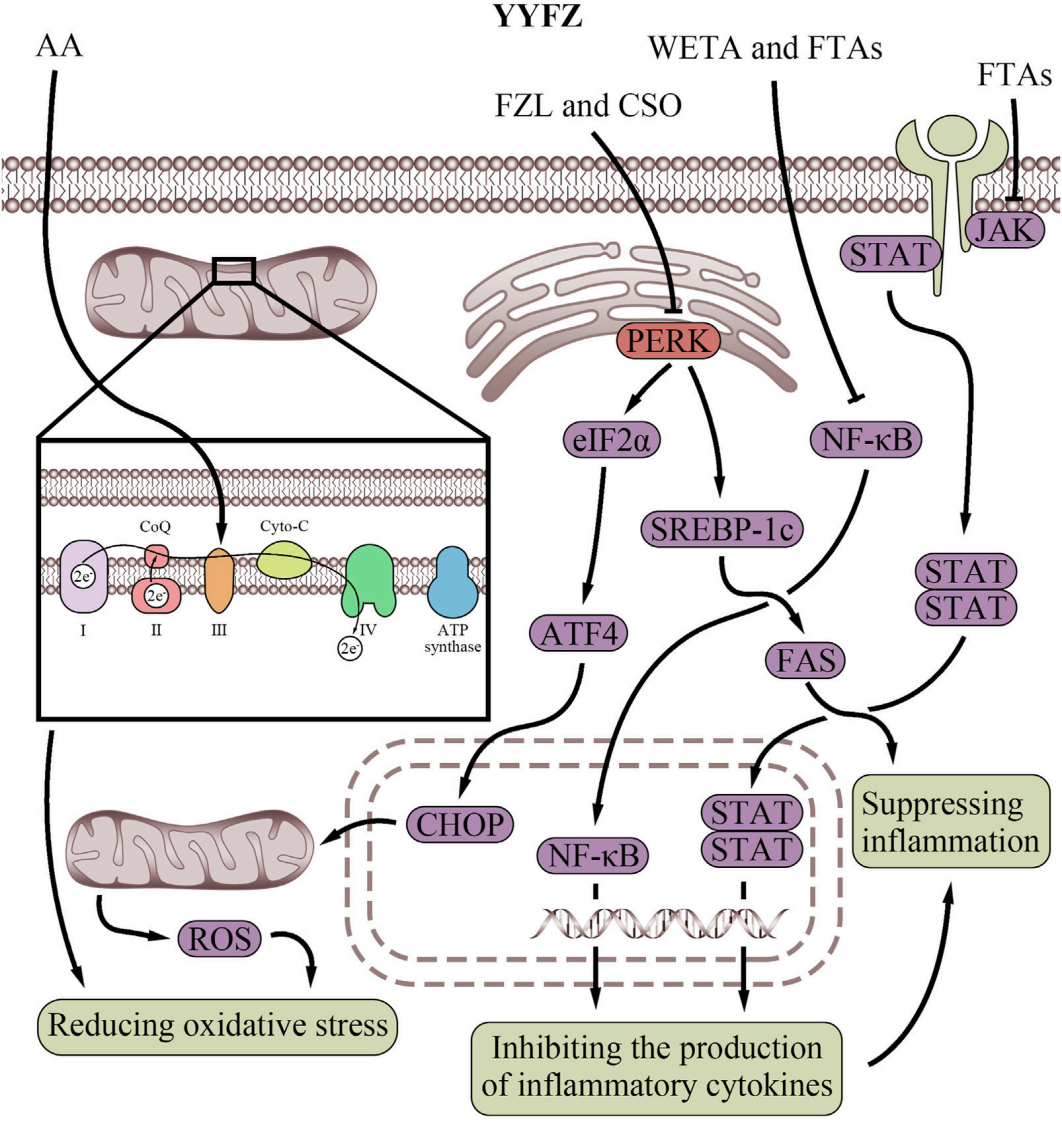
Antioxidant and anti-inflammatory effects of YiyiFuzi Powder’s major components (Abbreviations: AA, arachidonic acid; ATF4, activating transcription factor 4; ATP, adenosine triphosphate; CHOP, C/EBP homologous protein; Co Q, Coenzyme Q; CSO, Coix seed oil; Cyto-C, cytochrome C; eIF2α, eukaryotic initiation factor 2 alpha; FAS, fatty acid synthase; FPS, polysaccharides from Fuzi; FTAs, Fuzi total alkaloids; FZL, fuziline; JAK, Janus kinase; NF-κB, nuclear factor-kappa B; PERK, protein kinase RNA-like endoplasmic reticulum kinase; ROS, reactive oxygen species; SREBP-1c, sterol regulatory element-binding protein-1c; STAT, signal transducer and activator of transcription; WETA, water extraction of toasted *Aconitum carmichaelii* Debx.; YYFZ, YiyiFuzi Powder).

Coicis semen contains high levels of antioxidants, including phenolic compounds, flavonoids, gamma-aminobutyric acid, CSP, and polyphenols (Xu et al., 2017; Zeng et al., 2021; Fang et al., 2023; Lee et al., 2023; Zhao et al., 2023). These antioxidants help prevent cellular oxidative stress and mitochondrial oxidative damage (Table 4; Figure 5), protecting mitochondrial integrity (Brand et al., 2004) and function (Chen et al., 2022). CSO also contains small amounts of free FAs, including arachidonic acid (AA) (Chen et al., 2022). However, AA exerts different effects on the mitochondrial energy-conserving system depending on its concentration. At sub-micromolar concentrations, AA can rescue proton pumping by the complex III (bc1 complex) of the mitochondrial, ETC., (Di Paola and Lorusso, 2006). Bc1 complex plays a key role in transferring electrons from ubiquinol to cytochrome c (Cyto-C) while pumping protons, which belongs to the modified Q cycle pathway and is necessary for ATP synthesis (Alberts et al., 2002; Springett, 2021). However, impaired, ETC., increases mitochondrial ROS production. Acute ROS exposure shuts down mitochondrial energy production, while chronic ROS causes oxidative damage to mitochondrial and cellular proteins, lipids, and nucleic acids (Chen and Zweier, 2014). Meanwhile, at micromolar concentrations, AA can increase inner mitochondrial membrane proton conductance, acting as a protonophore (Di Paola and Lorusso, 2006). Increasing inner membrane proton conductance can protect the cardiovascular system by reducing ROS production and limiting mitochondrial damage (Cheng et al., 2017). This process also belongs to the modified Q cycle pathway (Di Paola and Lorusso, 2006). Therefore, the small amount of AA in CSO may enhance mitochondrial electron transport and reduce ROS production via the modified Q cycle pathway, thus protecting mitochondria and the cardiovascular system.

**TABLE 4:** Regulatory effects of YYFZ’s major components on oxidative stress and inflammatory response.

Herbal medicine	Components	Pathways/Targets	Potential effect
*Coix lacryma-jobi* L.var. *ma-yuen* Stapf	AA	Regulating the modified Q cycle pathway	Rescuing proton pumping by the complex III (bc1 complex) of the mitochondrial, ETC., and increasing inner mitochondrial membrane proton conductance to reduce ROS production and limit mitochondrial damage
*Aconitum carmichaelii* Debx.	FZL	Lowering GSDMD, 8-OHDG, IL-1β, and GAL-3	Reducing oxidative stress and helping maintain mitochondrial function
	FZL	Downregulating the PERK/eIF2α/ATF4/CHOP signaling pathway	Inhibiting ROS-triggered ER stress and ISO-induced mitochondrial oxidative stress injury and apoptosis, meanwhile maintaining MMP and restoring mitochondrial function
	berberine, rutin, and 5-caffeoylquinic acid	Inhibiting MAO-B activity	Reducing oxidative stress and improving mitochondria
	FPS	Enhancing MnSOD activity	Suppressing mitochondrial oxidative stress
*Coix lacryma-jobi* L.var. *ma-yuen* Stapf	CSO	Potentially suppressing the PERK/SREBP-1c/FAS signaling pathway	Improving systemic inflammation and protecting mitochondria
*Aconitum carmichaelii* Debx.	WETA	Inhibiting the nuclear factor NF-κB signaling pathway	Inhibiting immune cell infiltration into heart tissue and production of pro-inflammatory cytokines
	FZL	Reducing the levels of GSDMD, IL-1β, 8-OHDG, and GAL-3	Reducing inflammatory factor level
	FTAs	Inhibiting the NF-κB and JAK/STAT signaling pathways	Exerting anti-inflammatory effects

AA, arachidonic acid; ATF4, activating transcription factor 4; CHOP, C/EBP, homologous protein; CSO, coix seed oil; eIF2α, eukaryotic initiation factor 2 alpha; ETC., electron transport chain; FAS, fatty acid synthase; FPS, polysaccharides from Fuzi; FTAs, Fuzi total alkaloids; FZL, fuziline; GAL-3, Galectin-3; GSDMD, Gasdermin D; IL, interleukin; ISO, isoproterenol; JAK, janus kinase; MAO-B, monoamine oxidase B; MMP, mitochondrial membrane potential; MnSOD, manganese superoxide dismutase; NF-κB, nuclear factor-kappa B; PERK, protein kinase RNA-like endoplasmic reticulum kinase; ROS, reactive oxygen species; SREBP-1c, sterol regulatory element-binding protein-1c; STAT, signal transducer and activator of transcription; WETA, water extraction of toasted *Aconitum carmichaelii* Debx.; YYFZ, YiyiFuzi Powder; 8-OHDG, 8-hydroxydeoxyguanosine.

Antioxidants in Fuzi also help reduce oxidative stress (Table 4; Figure 5), protecting against mitochondrial and cardiovascular damage. A study found that FZL reduced dobutamine-induced cardiac damage in mice by lowering Gasdermin D (GSDMD), interleukin (IL)-1β, 8-hydroxydeoxyguanosine (8-OHDG), and Galectin-3 (GAL-3), and balancing oxidant and antioxidant levels, thereby reducing oxidative stress (Hacanli et al., 2023). Reducing oxidative stress helps maintain mitochondrial function and enhances left ventricular function (Lucas and Szweda, 1998). Research also showed that FZL can alleviate ISO-induced injury, both *in vitro* and *in vivo*, by inhibiting ROS-triggered ER stress via suppressing the PERK/eIF2α/ATF4/CHOP pathway (Fan et al., 2020). This pathway is a key ER stress response route, and its dysregulation can lead to cell death and cardiovascular disorders (Zhang et al., 2017). In this study, FZL also inhibited ISO-induced mitochondrial oxidative stress injury and apoptosis, maintained MMP, and restored mitochondrial function to play the cardiovascular protective role (Fan et al., 2020). Besides, Fuzi contains bioactive components (including berberine, rutin, and 5-caffeoylquinic acid) (Ayeni et al., 2023) that inhibit an enzyme, monoamine oxidase B, thereby reducing oxidative stress, improving mitochondria, and contributing to the enhancement of left ventricular function (Kaludercic et al., 2014). In addition, studies in SD rats have shown that FPS exerts mitochondrial and cardiomyocyte protective effects (Liu and Ji, 2011). FPS suppresses mitochondrial oxidative stress by enhancing the gene and activity expression of Mn-SOD, an important antioxidant enzyme. Additionally, FPS was demonstrated to inhibit cardiomyocyte apoptosis, reduce lipid peroxidation, and stabilize MMP function (Liu and Ji, 2011; Xing et al., 2023).

### 4.5 Inhibiting inflammatory responses

Inflammatory responses may lead to abnormal mitochondrial structure and function (López-Armada et al., 2013), as well as ER stress (Degechisa et al., 2022). Evidence also suggests that mitochondrial damage and ER stress can activate inflammation (Gao et al., 2020; Degechisa et al., 2022). However, reducing the inflammatory response has been linked to protecting both mitochondria and the ER, thereby contributing to cardiovascular protection (Liu et al., 2020).

The anti-inflammatory properties of YYFZ were demonstrated through clinical metabolomics, network pharmacology, molecular docking, and atomic force microscopy analysis (Wang, 2019; Wang Y. et al., 2023), via inhibiting the activation of the MAPK signaling pathways, reducing the production of TNF-α, and promoting the release of anti-inflammatory substances. This powder is commonly used in China to treat various inflammatory conditions such as arthritis and myocarditis (Liu, 1991; Zhang F. F. et al., 2023; Wang Y. Y. et al., 2023). Its anti-inflammatory effects are attributed to its active components, including flavonoids, stilbenes, and anthraquinones (Chung et al., 2010; Zhu, 2017; Feng et al., 2021; Sui and Xu, 2022).

Coicis semen possesses anti-inflammatory properties (Table 4; Figure 5) and helps protect against CVD, as chronic inflammation is a risk factor (Zeng et al., 2022). Coicis semen’s anti-inflammatory ingredients include polyphenols, coixol, polysaccharides, eriodictyol, ceramide, and p-coumaric acid. Eriodictyol, ceramide, and p-coumaric acid from the ethanol extraction of Coicis semen hull were demonstrated to exhibit anti-inflammatory properties by increasing NO and prostaglandin E2 production induced by lipopolysaccharides (Huang et al., 2009). Methanol extraction of Coicis semen also shows anti-inflammatory properties, including the inhibition of NO and superoxide production by activated macrophages (Seo et al., 2000). Furthermore, CSO significantly prevents obesity and improves systemic inflammation (Liu et al., 2019). CSO may exert cardiovascular protection by reducing obesity-induced inflammation and protecting mitochondria through the PERK/SREBP-1c/FAS signaling pathway, which regulates lipid metabolism and inflammation (Xue et al., 2005; Zhang and Kaufman, 2008; Han and Kaufman, 2016; Saltiel and Olefsky, 2017; Wang S. et al., 2021; Chen et al., 2022).

Fuzi can protect against HF via its anti-inflammatory effects *in vivo* (Liu X. et al., 2022). Specifically, Water extraction of toasted *Aconitum carmichaelii* Debx. inhibits immune cell infiltration into heart tissue and the production of pro-inflammatory cytokines, which cause cardiovascular damage and contribute to HF progression by inhibiting the activation of the nuclear factor-kappa B (NF-κB) signaling pathway (Xing et al., 2023). FZL was also found to reduce the levels of inflammatory factors, such as GSDMD, IL-1β, 8-OHDG, and GAL-3, in mice with dobutamine-induced heart damage (Hacanli et al., 2023). Fuzi total alkaloids (FTAs) exert anti-inflammatory effects by promoting apoptosis, inhibiting hyperactivity of the NF-κB and Janus kinase/signal transducer and activator of transcription signaling pathways, and downregulating proliferation of tumor necrosis factor -α-induced MH7A cells (Wu et al., 2022). They also regulate related proteins of the mitochondrial apoptosis signaling pathway. This suggests that Fuzi’s anti-inflammatory properties are also mediated by mitochondria. Moreover, inflammation can affect Ca^2+^ flux between the ER and mitochondria, which is crucial for energy production and cell death. During inflammation, Ca^2+^ overload can cause mitochondrial damage, further activating the NLRP3 inflammasome (Giorgi et al., 2018). By mitigating inflammation, Fuzi safeguards mitochondrial and ER functions, ensuring balanced Ca^2+^ transport between these two organelles, and thereby exerting cardiovascular protection (Table 4; Figure 5).

### 4.6 Regulating cell autophagy and apoptosis

**FIGURE 6:**
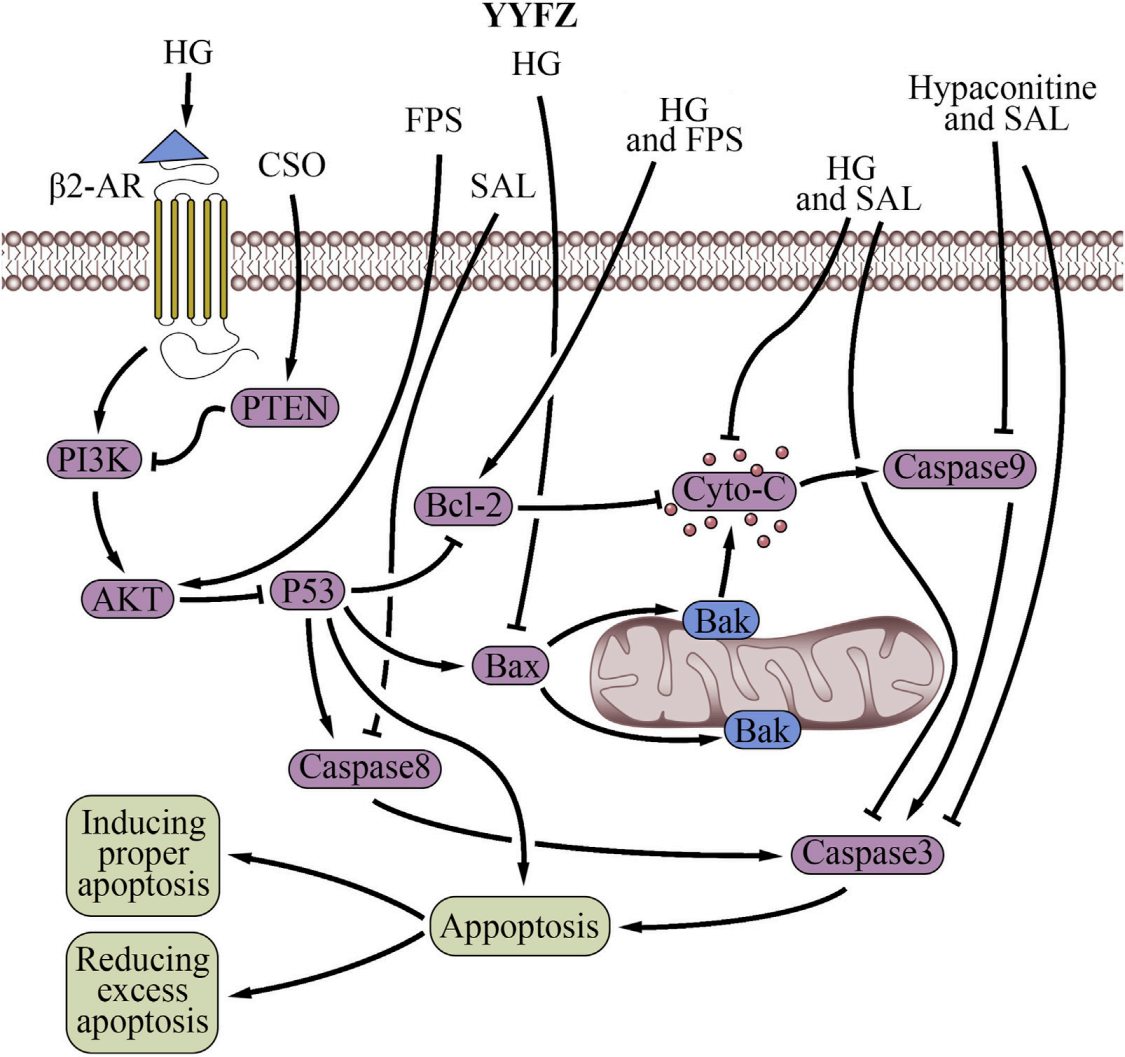
Cell apoptosis regulatory effects of YiyiFuzi Powder (Abbreviations: AMPK, AMP-activated protein kinase; β2-AR, β2-adrenergic receptors; CSO, Coix seed oil; Cyto-C, cytochrome C; FPS, polysaccharides from Fuzi; HG, higenamine; PI3K, phosphoinositide 3-kinase; PTEN, phosphatase and tensin homolog; SAL, salsolinol; YYFZ, YiyiFuzi Powder).

While the specific mechanism of action of YYFZ in regulating mitochondrial apoptosis is not fully understood, research suggests that it may involve multiple pathways (Table 5; Figure 6). One possible way is to modulate the balance of pro-apoptotic and anti-apoptotic protein expression. YYFZ may interact with Caspases, Bcl-2 family proteins, and the MAPK signaling pathways (Wang, 2019; Wang Y. et al., 2023; Wang Y. Y. et al., 2023). YYFZ can also indirectly protect cells from apoptosis-induced oxidative stress, for it scavenges ROS and reduces oxidative stress (Wang, 2019; Wang Y. et al., 2023; Wang Y. Y. et al., 2023). In addition to the above mechanisms, network pharmacology analysis showed that YYFZ acts through the PI3K/AKT, Ras(Wang Y. et al., 2023), and cAMP signaling pathway (Wang, 2019), with direct or indirect effects on interactions of mitochondria and ER.

**TABLE 5:** Regulatory effects of YYFZ’s major components on cell apoptosis and calcium homeostasis.

Herbal medicine	Components	Pathways/Targets	Potential effect
*Coix lacryma-jobi* L.var. *ma-yuen* Stapf	—	Activating the AMPK/JNK signaling pathway	Inhibiting mitochondrial-dependent apoptosis
	Ethanol CSE	Activating the IRE1 and the PERK signaling pathways thereby stimulating the UPR pathway	Increasing the synthesis of CHOP then inducing apoptosis
	CSO	Activating the PTEN/PI3K/AKT signaling pathway	Inducing apoptosis
*Aconitum carmichaelii* Debx.	FZL	Suppressing the mitochondrial apoptosis pathway	Reducing apoptosis
	FPS	Promoting expression of Bcl-2	Scavenging excess mitochondrial oxygen radicals and inhibiting apoptosis
	FPS	Activating the AKT signaling pathway	Reducing apoptosis
	FPS	Activating the AMPK/mTOR signaling pathway	Induce autophagy
	Hypaconitine	Decreasing Caspase 3 and Caspase 9 expression	Reducing apoptosis of myocardial cells
	SAL	Decreasing Cyto-C, Caspase 3, Caspase 8, and Caspase 9 expressions, while increasing the Bcl-2 expression	Reducing adriamycin-induced myocardial injury and apoptosis
	HG	Decreasing Caspase 3 activity, Bax expression, and Cyto-C release, while increasing Bcl-2 expression	Attenuating apoptosis
	HG	Suppressing AMPK activation	Attenuating myocyte apoptosis
	HG	Activating the β2-AR/PI3K/AKT signaling pathway	Attenuating apoptosis
	FTAs	Regulating the mitochondrial apoptosis signaling pathway	Promoting apoptosis
*Coix lacryma-jobi* L.var. *ma-yuen* Stapf	Naringenin	Potentially interacting with Ca^2+^ signaling pathway	Decreasing uterine contraction and intracellular Ca^2+^ concentrations by blocking extracellular Ca^2+^ influx
	Quercetin	Potentially interacting with Ca^2+^ signaling pathway	Decreasing uterine contraction and intracellular Ca^2+^ concentrations by blocking extracellular Ca^2+^ influx
*Aconitum carmichaelii* Debx.	SAL	Inhibiting the MCU signaling pathway	Maintaining intracellular calcium homeostasis
	SAL	Reducing MCU, MICU1, and MICU2 levels	Maintaining intracellular calcium homeostasis

AMPK, AMP-activated protein kinase; β2-AR, β2-adrenergic receptors; CHOP, C/EBP, homologous protein; CSE, coix seed extract; CSO, coix seed oil; FZL, fuziline; ER, endoplasmic reticulum; Cyto-C, cytochrome C; FPS, polysaccharides from Fuzi; FTAs, Fuzi total alkaloids; HG, higenamine; IRE1, inositol-requiring enzyme 1; JNK, c-Jun N-terminal kinase; MCU, mitochondrial calcium uniporter; MICU, mitochondrial calcium uptake; mTOR, mechanistic target of rapamycin; PERK, protein kinase RNA-like ER, kinase; PI3K, phosphoinositide 3-kinase; PTEN, phosphatase and tensin homolog; SAL, salsolinol; UPR, unfolded protein response; YYFZ, YiyiFuzi Powder.

The mitochondria-mediated apoptotic pathway, known as the intrinsic pathway, is a major pathway that induces apoptosis (Ding et al., 2012), which is a critical mechanism for programmed cell death. Prolonged ER stress or adaptive response failure also leads to apoptotic cell death (Szegezdi et al., 2006). Both ER stress and mitochondria-induced apoptosis are involved in physiological and pathological processes. Nuanxin capsules containing Coicis semen had a protective effect on HF which enhanced cardiac function by inhibiting mitochondrial-dependent apoptosis through the AMPK/c-Jun N-terminal kinase signaling pathway under oxidative stress (Lou et al., 2021). FZL prevented cardiac myocyte necrosis (Hacanli et al., 2023). It reduced ER stress-induced apoptosis and improved cardiac function in rats with ISO-induced injury by suppressing the mitochondrial apoptosis pathway (Fan et al., 2020).

FPS inhibited apoptosis in a myocardial I/R injury cell model by promoting the expression of the anti-apoptotic gene Bcl-2 and scavenging excess mitochondrial oxygen radicals (Liu and Ji, 2011; Xing et al., 2023). Studies showed that Fuzi decreases Caspase three and Caspase nine expression to prevent unwanted activation of the mitochondrial apoptotic pathway in healthy cardiac cells (Wang and Youle, 2009). Caspase nine is an initiator caspase that activates the executioner caspase, Caspase 3, which then carries out the process of apoptosis (Mondello et al., 2021). The anti-apoptotic effects through the mitochondrial apoptosis signaling pathway are also associated with the decrease of Caspase 8, Cyto-C, Fas, and Bax expression, and the increase of Bcl-2 expression. Decreasing Caspase 8, Cyto-C, and Fas expression can potentially protect cardiac cells from apoptosis and mitigate various cardiac pathologies, including MI, cardiomyopathy, and HF (van Empel et al., 2005; Cao et al., 2023). Furthermore, Bax is a proapoptotic protein that promotes cell death, while Bcl-2 is an antiapoptotic protein that inhibits cell death. Fang, et al. reported that HA extracted from Fuzi at a concentration of 250 ng/mL reduces the H2O2-induced apoptosis of myocardial cells (Fang et al., 2010). They hypothesized that the mechanism probably involves the decrease in Caspase three and Caspase nine expression and the increase in cell proliferation activity (Fang et al., 2010). According to Wen et al., SAL in Fuzi could reduce adriamycin-induced myocardial injury and apoptosis in acute exhausted SD rats (Wen et al., 2019b). This study found that SAL decreased the expressions of Fas, Cyto-C, Caspase 3, Caspase 8, and Caspase 9, and increased the expression of Bcl-2. Earlier study found that the administration of HG significantly decreased Caspase three activity, Bax expression, and Cyto-C release, while upregulated Bcl-2 expression before rats underwent I/R injury (Lee et al., 2006). Furthermore, research has indicated that HG can attenuate DOX-induced cardiac remodeling and myocyte apoptosis by suppressing AMPK activation (Jin et al., 2022). Another in-depth study found that HG counteracted apoptosis and protected against I/R-induced MI in rat cardiomyocytes by activating β2-AR and the β2-AR/PI3K/AKT signaling pathway (Wu M. P. et al., 2016). Upon activation of the AKT pathway, antiapoptotic proteins such as Bcl-2 and Bcl-xL are upregulated, preventing the translocation of proapoptotic factors like Bad and Bax to mitochondria (Davidson et al., 2020). Previous studies have demonstrated that FPS treatment results in increased expression of the antiapoptotic protein Bcl-2 using an *in-vitro* model of myocardial I/R injury. This suggests FPS may promote cardiomyocyte survival by reducing apoptosis through Bcl-2-mediated mechanisms via the AKT pathway (Liu et al., 2012). The Mahuang Xixin Fuzi decoction inhibited apoptosis through the mitochondrial pathway by ameliorating MMP reduction, blocking mitochondrial Cyto-C release, reducing Bax level, increasing Bcl-2 level, and suppressing Caspase nine and Caspase three activation (Yang et al., 2022b).

However, the regulatory effect of YYFZ on apoptosis is bidirectional. Ethanol extract of Coicis semen was shown to stimulate the UPR or induce ER stress via activation of the IRE1 and PERK signaling pathways in normal liver cells while inducing apoptosis via increasing the synthesis of CHOP through the UPR pathway (Szegezdi et al., 2006; Oslowski and Urano, 2011; Kim et al., 2018). An *in-vitro* study demonstrated that CSO upregulates the PTEN protein while inhibiting the expression of p-AKT and p-PI3K proteins, thereby inducing apoptosis in human pancreatic cancer cells through the PTEN/PI3K/AKT signaling pathway (Yang et al., 2022a). Additionally, Coicis semen has been found to inhibit proliferation and promote the apoptosis of various cancer cell types, including cancer cells of the lung (Chang et al., 2003), colon (Lee et al., 2008), hepatic (Qu et al., 2016), mouth (Manosroi et al., 2019), breast (Fang et al., 2020; Chiang et al., 2022), uterine (Huang et al., 2021), cervical (Chen et al., 2018; Guo et al., 2019; Chiang et al., 2022), and pancreatic (Yang et al., 2022a). While not directly related to cardiovascular health, this could contribute to overall health and wellbeing (Zeng et al., 2022). Fuzi also possesses the ability to promote apoptosis. FTAs promoted apoptosis by regulating the mitochondrial apoptosis signaling pathway and its related proteins *in vitro* (Wu et al., 2022). Li et al. demonstrated that FPS induces autophagy and attenuates starvation-induced cardiomyocyte (H9c2 cells) death through the AMPK/mTOR pathway activation. AMPK helps restore energy supply and mitochondrial function by promoting ATP production in the failing heart (Liao et al., 2013). In summary, YYFZ has bidirectional effects on mitochondrial apoptosis regulation. The effect of YYFZ inhibits apoptosis mainly through suppressing the UPR and mitochondrial apoptosis signaling pathway, while the promotion of apoptosis occurs via the activation of the IRE1, PERK, UPR, and AMPK/mTOR signaling pathways.

### 4.7 Calcium homeostasis

Mitochondrial Ca^2+^ homeostasis plays a vital role in cardiomyocyte energy metabolism and function (Zhang D. et al., 2022). Ca^2+^ overload damages mitochondria and impairs mitochondrial function. Wang et al. demonstrated that YYFZ regulates Ca^2+^ by modulating the expression and activity of calcium channels and transporters in a network pharmacology analysis (Wang, 2019). Previous studies have shown that ethyl acetate CSEs decreased uterine contraction and intracellular Ca^2+^ concentrations by blocking extracellular Ca^2+^ influx in rats (Zeng et al., 2022). Naringenin and quercetin have been identified as major constituents present in these extracts. This suggests naringenin and quercetin may interact with calcium channels or other elements of the Ca^2+^ signaling pathway, thereby influencing Ca^2+^ homeostasis. In HF, abnormal Ca^2+^ handling is characterized by elevated end-diastolic cytosolic Ca^2+^ levels and prolonged calcium transients during diastole (Gorski et al., 2015). Therefore, blocking Ca^2+^ influx could help restore balance and improve cardiac function. Further research is still needed to determine whether Coicis semen regulates Ca^2+^ homeostasis to prevent CVD.

The MCU on the inner membrane is primarily responsible for mitochondrial Ca^2+^ uptake due to its high selectivity and low affinity for Ca^2+^(Kirichok et al., 2004). The MCU, along with mitochondrial calcium uptake 1 (MICU1) and MICU2, are key components of the MCU signaling pathway, regulating cardiomyocyte function by controlling mitochondrial Ca^2+^ uptake (Mammucari et al., 2018; Wen et al., 2019b). By inhibiting the overactivation of the MCU signaling pathway, SAL can reduce adriamycin-induced myocardial injury and apoptosis (Wen et al., 2019b). This improves mitochondrial energy metabolism and cardiac function by reducing MCU, MICU1, and MICU2 levels both in mRNA and protein levels, controlling Ca^2+^ transport, and maintaining intracellular Ca^2+^ homeostasis (Wen et al., 2019b). Shenfu injection (SFI), a TCM containing Fuzi, has been used in treating CVD for a long time in China (Committee, 2005). Appropriate doses of SFI decreased Ca^2+^ overload and protected myocardial mitochondria in an I/R rabbit model (Cao et al., 2005). Moreover, inflammation can impact Ca^2+^ flux between the ER and mitochondria, which is crucial for energy production and cell death. During inflammation, mitochondrial damage from Ca^2+^ overload can further enhance NLRP3 inflammasome activation (Giorgi et al., 2018). Fuzi has demonstrated anti-inflammatory effects that may safeguard against CVD by helping maintain balanced Ca^2+^ transport between mitochondria and ER to prevent mitochondrial damage (Table 5; Figure 3).

## 5 Toxicity

Coicis semen has been demonstrated to promote apoptosis of cancer cells to maintain homeostasis, and Fuzi also possesses this ability, as mentioned earlier. Therefore, indiscriminate use of YYFZ may also induce cardiomyocyte apoptosis, potentially damaging the myocardium. Besides, Coicis semen treatment has been found to promote angiogenesis in ischemic stroke models. This effect is mediated through the transforming growth factor-beta (TGF-β)/ALK1/Smad1/5 signaling pathway (Du et al., 2020). However, in the context of atherosclerosis, it has been suggested that the balance between Smad1/5- and Smad2/3-dependent signaling determines the outcome of the effect of TGF-β. Smad2/3 regulates the atheroprotective effects, whereas Smad1/5 is responsible for the proatherogenic effects of TGF-β(Yuan and Jing, 2010). Furthermore, in a study on mouse podocytes, TGF-β1 was found to increase mitochondrial OCR and ATP generation (Abe et al., 2013), which may exacerbate myocardial hypoxia.

The expression of PGC-1α and mitochondrial biogenesis can be suppressed by AC. This eventually leads to apoptosis in H9c2 cardiac cells, thereby demonstrating the toxic side effects of aconitum (Gao et al., 2018). PGC-1α plays a significant role in promoting mitochondrial biosynthesis (Fontecha-Barriuso et al., 2020; Abu Shelbayeh et al., 2023). A series of changes after PGC-1α expression reduction, such as a significant decrease in Bcl-2/Bax ratio and an increase in the levels of Cyto-C and Caspase 3, ultimately led to apoptosis in H9C2 cells (Gao et al., 2018).

Another *in-vitro* study found that AC treatment significantly reduced the phosphorylation of AMPK in SH-SY5Y cells. This suggests that certain doses of Fuzi may suppress the AMPK signaling pathway. Consequently, by inhibiting the AMPK signaling pathway, Fuzi could induce mitochondrial energetic and metabolic dysfunction with aberrant mitochondrial dynamics in cells (Yang et al., 2021a). This research highlights the toxicity of Fuzi, and overdosage in clinical practice warrants prudence. Nevertheless, Fuzi has been found to have a synergistic effect and reduce its toxicity when used in combination with other TCM(Liu X. et al., 2022). It can be inferred that the combination of Coicis semen and Fuzi may strengthen the therapeutic effect and reduce toxicity. However, due to their known toxicity, caution must be exercised when clinically employing YYFZ, and more importantly, under the guidance of a healthcare professional.

## 6 Conclusion

YYFZ, an effective TCM prescription for CVD, improves clinical symptoms, alleviates myocardial damage, enhances myocardial function, and reduces cardiovascular risk factors. Its mechanisms are associated with antioxidant, anti-inflammatory, immunomodulatory, regulation of cell proliferation and apoptosis, and modulation of mitochondrial and ER functions, reflecting the multicomponent, multitarget, and multi-pathway properties of TCM. However, due to the known toxicity of Coicis semen and Fuzi, caution should be exercised when using YYFZ, and it should be administered under the guidance of professional TCM practitioners. More rigorous experimental research and high-quality clinical studies are needed in the future to confirm these findings.

## Figures and Tables

**Table:** 

**AA**	arachidonic acid
**AC**	Aconitine
**AMPK**	AMP-activated protein kinase
**ATF4**	activating transcription factor 4
**ATP**	adenosine triphosphate
**ATP5A1**	ATP synthase
**H+ transporting**	mitochondrial F1 complex, alpha subunit 1
**BAC**	benzoylaconine
**β-AR**	β-adrenergic receptors
**BCAAs**	branched-chain amino acids
**cAMP**	cyclic adenosine monophosphate
**CHD**	coronary heart disease
**CHF**	chronic heart failure
**CHOP**	C/EBP homologous protein
**CSE**	Coix seed extract
**CSO**	Coix seed oil
**CSP**	Coix seed polysaccharides
**CVD**	cardiovascular diseases
**Cyto-C**	cytochrome c
**DCM**	diabetic cardiomyopathy
**DOX**	doxorubicin
**eIF2α**	eukaryotic initiation factor 2 alpha
**ER**	endoplasmic reticulum
**ERAD**	endoplasmic reticulum-associated protein degradation
**FA**	fatty acids
**FAS**	fatty acid synthase
**FPS**	polysaccharides from Fuzi
**FTAs**	Fuzi total alkaloids
**FZL**	Fuziline
**GAL-3**	Galectin-3
**GSDMD**	Gasdermin D
**HA**	hypaconitine
**HF**	heart failure
**HG**	higenamine
**HPLC**	high-performance liquid chromatography
**IGF-1**	insulin-like growth factor 1
**IL**	interleukin
**IRE1**	inositol-requiring enzyme 1
**ISO**	isoproterenol
**I/R**	ischemia/reperfusion
**LDL-C**	low-density lipoprotein cholesterol
**LKB1**	liver kinase B1
**MA**	mesaconitine
**MAM**	mitochondria-associated endoplasmic reticulum membranes
**MAPK**	modulating mitogen-activated protein kinase
**MCU**	mitochondrial calcium uniporter
**MI**	myocardial infarction
**MICU**	mitochondrial calcium uptake
**MMP**	mitochondrial membrane potential
**Mn-SOD**	manganese superoxide dismutase
**MQC**	mitochondrial quality control
**MS**	mass spectrometry
**mTOR**	mammalian target of rapamycin
**NF-κB**	nuclear factor-kappa B
**NO**	nitric oxide
**OCR**	oxygen consumption rate
**OPA1**	optic atrophy 1
**OXPHOS**	oxidative phosphorylation
**PERK**	protein kinase RNA-like endoplasmic reticulum kinase
**PGC-1α**	peroxisome proliferator-activated receptor γ coactivator 1α
**PINK1**	PTEN-induced putative kinase 1
**PPAR**	peroxisome proliferator-activated receptor
**PI3K**	phosphoinositide 3-kinase
**PKA**	protein kinase A
**PTEN**	phosphatase and tensin homolog
**QLQX**	Qili Qiangxin Capsule
**Q-TOF**	Quadrupole Time-of-Flight
**ROS**	reactive oxygen species
**SAL**	salsolinol
**SFI**	Shenfu injection
**SIRT**	Sirtuin
**SREBP-1c**	sterol regulatory element-binding protein-1c
**TC**	total cholesterol
**TCM**	Traditional Chinese medicine
**TG**	triglycerides
**TGF-β**	transforming growth factor-beta
**UPR**	unfolded protein response
**YYFZ**	YiyiFuzi Powder
**ZR**	Zingiberis Rhizoma
**8-OHDG**	8-hydroxydeoxyguanosine
